# Unrecognized Dengue Virus Infections in Children, Western Kenya, 2014–2015

**DOI:** 10.3201/eid2311.170807

**Published:** 2017-11

**Authors:** David M. Vu, Noah Mutai, Claire J. Heath, John M. Vulule, Francis M. Mutuku, Bryson A. Ndenga, A. Desiree LaBeaud

**Affiliations:** Stanford University School of Medicine, Stanford, California, USA (D.M. Vu, C.J. Heath, A.D. LaBeaud);; Kenya Medical Research Institute, Centre for Global Health Research, Kisumu, Kenya (N. Mutai, B.A. Ndenga);; Technical University of Mombasa, Mombasa, Kenya (J.M. Vulule, F.M. Mutuku)

**Keywords:** dengue, Kenya, children, disease outbreaks, flavivirus, arbovirus infection, vector-borne infections, viruses, dengue viruses

## Abstract

We detected a cluster of dengue virus infections in children in Kenya during July 2014–June 2015. Most cases were serotype 1, but we detected all 4 serotypes, including co-infections with 2 serotypes. Our findings implicate dengue as a cause of febrile illness in this population and highlight a need for robust arbovirus surveillance.

Due to lack of national surveillance programs, the extent of infection with dengue viruses (DENV) among children is largely unknown in much of sub-Saharan Africa (*1*). Uncovering this hidden burden is critical for making informed public health decisions that affect populations that are most vulnerable to vectorborne disease. To address this knowledge gap, in January 2014, we initiated a 4.5-year study of the transmission and extent of arbovirus infection in children in Kenya. Although the study continues through June 2018, we identified a cluster of DENV infections among febrile children in western Kenya, prompting this report to raise awareness of DENV as a cause of acute febrile illness (AFI) among children in this country.

We describe results from a cohort of children with AFI who came to 1 of 2 regional health centers that serve the communities of Chulaimbo (a rural village) and Kisumu (an urban city), both located in western Kenya. The cohort is part of a larger ongoing parent study that enrolls additional child cohorts at other study sites or with other study designs and collects vector and environmental data. Details of the parent study are beyond the scope of this report and will be described elsewhere. The study is being conducted under the supervision of the institutional review board of Stanford University (Stanford, CA, USA; IRB-31488) and the scientific and ethics review unit of the Kenya Medical Research Institute (Kisumu, Kenya; SSC 2611). 

For this study, we enrolled febrile children 1–17 years of age. At the enrollment and 1-month follow-up visits, we collected information on demographics and risk factors, performed a physical examination, and obtained blood samples. We tested the blood samples for DENV RNA by reverse transcription PCR (RT-PCR) (*2*).

We enrolled 1,258 children with AFI during January 2014–April 2016. We tested blood samples from 1,104 study participants (87.8%) by RT-PCR. Of those, 82 (7.4%) were positive for DENV RNA: 58 (70%) were serotype 1 (DENV-1), 2 (2.4%) were serotype 2 (DENV-2), 13 (15.9%) were serotype 3 (DENV-3), and 2 (2.4%) were serotype 4 (DENV-4) (Figure). We also detected co-infection with 2 serotypes in 6 participants: 2 children had DENV-1 and -3; 3 children had DENV-1 and -4; and 1 child had DENV-2 and -3.

**Figure:**
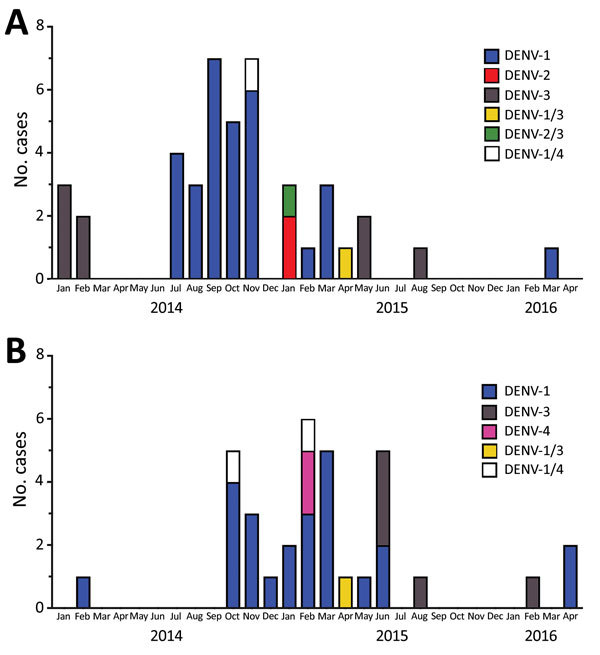
Epidemic curve for DENV infection in children in 2 locations in western Kenya, 2014–2016. A) Chulaimbo, a rural village; B) Kisumu, an urban city. Serotypes are indicated; some children were infected with multiple serotypes. DENV, dengue virus.

Most DENV cases occurred during July 2014–June 2015. The likelihood of testing positive for DENV by PCR did not differ by age or sex. The median age of DENV PCR-positive participants was 3.5 years (IQR 2.0–5.2 years); 43.9% of participants were female. We did observe a higher likelihood of testing positive by PCR for children from the rural site, Chulaimbo (9.6%), compared with those from the urban site, Kisumu (5.8%) (p = 0.017 by Fisher exact test; odds ratio 1.97, 95% CI 1.14–3.43, adjusted for age and sex). Continued surveillance is needed to investigate whether DENV is endemic to these areas and if there are differences in regional endemicity.

Although reports of DENV infection in Kenya remain sparse, several sero-epidemiologic studies have produced evidence of DENV transmission by demonstrating serum DENV IgG in patients of all ages (*3*–*6*). In 2013, an outbreak of DENV-2 in Mombasa was detected, in part, by RT-PCR (*7*). More recently, DENV infections were described in 43 adult patients with fever who resided in Mtwapa, on the coast of Kenya near Mombasa; these cases occurred February 2014–January 2015 (*8*), which overlapped with the period in which we observed the DENV cases we report in this study. All of the DENV PCR-positive subjects from the 2016 study were infected with DENV-2, which is consistent with the DENV serotype observed in the 2013 Mombasa outbreak (*7*). In contrast, most DENV PCR-positive subjects from our study were infected with DENV-1, which suggests regional variation in DENV strain predominance.

DENV viremia during acute infection is known to be transient (*9*); thus, our definition of DENV cases, based on RT-PCR detection of RNA, probably underestimated the true incidence of DENV infection. Data from testing of serum samples for DENV IgM and IgG seroconversion were not available as we wrote this report but would provide confirmation of our PCR results by a second method of detecting DENV infection. IgM testing, although less specific than PCR (*10*), may identify additional cases of DENV infection and could alter the descriptive statistics we present by enhancing our sensitivity for detecting DENV infection.

Improving efforts to detect DENV infections will raise awareness of DENV and increase the likelihood that healthcare providers will suspect it in patients with AFI. Correct diagnoses improve the ability of public health ministries to detect and react to outbreaks of DENV or other arboviral illnesses, rather than contributing to cycles of missed opportunities for preventive interventions.
